# Generalized Filter Bank Orthogonal Frequency Division Multiplexing: Low-Complexity Waveform for Ultra-Wide Bandwidth and Flexible Services

**DOI:** 10.3390/e26110994

**Published:** 2024-11-18

**Authors:** Yu Xin, Jian Hua, Tong Bao, Yaxing Hao, Ziheng Xiao, Xin Nie, Fanggang Wang

**Affiliations:** 1 State Key Laboratory of Mobile Network and Mobile Multimedia Technology, Shenzhen 518055, China; xin.yu@zte.com.cn (Y.X.); bao.tong@zte.com.cn (T.B.); 2 ZTE Corporation, Nanshan District, Shenzhen 518055, China; 3 State Key Laboratory of Advanced Rail Autonomous Operation, School of Electronic and Information Engineering, Beijing Jiaotong University, Beijing 100044, China; yaxinghao@bjtu.edu.cn (Y.H.); 23120134@bjtu.edu.cn (Z.X.); 23125045@bjtu.edu.cn (X.N.)

**Keywords:** 6G, terahertz, waveform, ultra-wide bandwidth

## Abstract

Terahertz (THz) communication is a crucial technique in sixth generation (6G) mobile networks, which allow for ultra-wide bandwidths to enable ultra-high data rate wireless communication. However, the current subcarrier spacing and the size of fast Fourier transform (FFT) of the orthogonal frequency division multiplexing (OFDM) in 5G NR are insufficient regarding the bandwidth requirements of terahertz scenarios. In this paper, a novel waveform is proposed to address the ultra-wideband issue, namely the generalized filter bank orthogonal frequency division multiplexing (GFB-OFDM) waveform. The main advantages are summarized as follows: (1) The *K*-point IFFT is implemented by two levels of IFFTs in smaller sizes, i.e, performing *M*-point IFFT in *N* times and performing *N*-point IFFT in *M* times, where K=N×M. (2) The proposed waveform can accommodate flexible subcarrier spacings and different numbers of the subbands to provide various services in a single GFB-OFDM symbol. (3) Different bandwidths can be supported using a fixed filter since the filtering is performed on each subband. In contrast, the cyclic prefix orthogonal frequency division multiplexing (CP-OFDM) in 4G/5G requires various filters. (4) The existing detection for CP-OFDM can be directly employed as the detector of the proposed waveform. Lastly, the comprehensive simulation results demonstrate that GFB-OFDM outperforms CP-OFDM in terms of the out-of-band leakage, complexity and error performance.

## 1. Introduction

The development of the sixth generation (6G) wireless communication system has been accompanied by the introduction of new technologies, among which terahertz (THz) technology, also referred to as one of the ten technologies that will innovate the future world, is a breakthrough spectrum technology [1,2]. It offers a wide transmission bandwidth to cater to the increasing demand for higher transmission rates, and thus it has emerged as a crucial component in 6G communication.

Terahertz communication typically involves the utilization of the electromagnetic waves within a frequency range spanning from 0.1 THz to 10 THz and a wavelength range from 3 mm to 30 μm. Its wavelength lies between the microwave and the far-infrared regions, providing an abundance of spectrum resources that remain unexplored [3]. One of the characteristics of the THz communication is ultra-wide bandwidth. Due to the abundant spectrum of resources brought by the very high frequency, the expected bandwidth of THz communication ranges from several GHz to tens of GHz, surpassing that of the current 5G bandwidth. Therefore, it can achieve an ultra-high rate of up to 1 Tbps, which is 10 to 100 times of the current 5G peak rate [4]. The other characteristics encompass remarkable penetrability [5], the capacity to traverse non-metallic materials without inflicting any damage, and the low energy levels that minimize harm to living organisms, thereby facilitating non-destructive testing. Furthermore, it provides finer time resolution for capturing subtle momentary forms and ensures utmost confidentiality.

In THz communication, achieving an ultra-wide bandwidth involves the following challenges. Hardware cost and implementation complexity set limitations for the maximum subcarrier spacing and the size of the fast Fourier transform (FFT) of orthogonal frequency division multiplexing (OFDM), which may hinder the utilization of the ultra-wide bandwidth in THz scenarios. Currently, the maximum subcarrier spacing is limited to 960 kHz and the maximum FFT size is restricted to 4096 points, resulting in a bandwidth limitation of around 4 GHz when considering oversampling and guard intervals. To support a larger bandwidth in OFDM baseband processing, there are typically two solutions, i.e., increasing the subcarrier spacing or the FFT size. However, increasing the subcarrier spacing leads to a shorter symbol duration, while a sufficiently long cyclic prefix is still required to combat multipath delay. This solution results in a higher proportion of cyclic prefix overhead and reduces the spectral efficiency. Typically, the complexity of *N*-point IFFT is NlogN [6]. The current hardware faces challenges in regard to accommodating the large size of the FFT. Therefore, it prompts us to propose a new waveform that enables the realization of ultra-wideband THz communication.

The waveforms predominantly employed in the current fifth generation (5G) standards encompass cyclic prefix-orthogonal frequency division multiplexing (CP-OFDM) and discrete Fourier transform spread orthogonal frequency division multiplexing (DFT-s-OFDM). In recent years, a plethora of investigations have been conducted on waveforms [7], including some new waveforms based on OFDM: (1) Filter-bank multi-carrier (FBMC) [8] is a form of real number modulation that separates the real and imaginary components of the modulated symbols, utilizing filters to achieve real orthogonality. (2) Generalized frequency division multiplexing (GFDM) [9,10] adopts a cyclic filter for each OFDM subcarrier and introduces block modulation technology; however, there is interference between subcarriers. (3) Filter bank orthogonal frequency division multiplexing (FB-OFDM) [11,12,13] utilizes polyphase filters for each subcarrier, with the subcarriers being approximately orthogonal. (4) Universal filtered multi-carrier (UFMC) [14] performs filtering on a set of consecutive subcarriers. Due to the long time-domain filter function, there is inter-symbol interference in the time domain. (5) Filter orthogonal frequency division multiplexing (F-OFDM) [15] filters the entire subband. (6) Orthogonal time frequency space (OTFS) [16,17,18,19] is a waveform based on a specific two-dimensional Fourier transform, i.e., a symplectic Fourier transform, which loads the modulated symbols into the delay-Doppler domain. These waveforms encounter various problems when supporting the communication in large bandwidths of the THz communication band.

Recently, we proposed a new waveform called generalized filter bank orthogonal frequency division multiplexing (GFB-OFDM) in [20,21,22] for THz communication. The waveform utilizes the two-level IFFTs and the polyphase filters to jointly process of the multiple subbands. In [21], it shows that the out-of-band leakage of GFB-OFDM is lower than that of CP-OFDM without error performance loss. In [22], more comparisons were provided in different subcarrier spacings. However, there is still a lack of solid proof regarding an equivalence between the GFB-OFDM transmitter and the CP-OFDM transmitter. Moreover, the complexity analysis and a more detailed evaluation are required to validate its advantages.

In this paper, we present the complexity analysis, solid proof of the equivalence between the two transmitters, and a comprehensive performance evaluation of the coded GFB-OFDM. By decomposing the large size of an IFFT into two levels of IFFTs in smaller sizes, the GFB-OFDM facilitates efficient handling of the large IFFT size and the subband filtering, which reduces the computational complexity and enables the ultra-wideband communication. The GFB-OFDM allows for a flexible configuration of the system parameters, including the number of subbands, the number of subcarriers in each subband, the subcarrier spacing, and the filter parameters, etc. This capability can improve the transmission stability in high-mobility scenarios by suppressing the effects of Doppler spread and satisfy various application requirements in 6G communication, especially in terms of the high bandwidth and different services. Furthermore, the GFB-OFDM can be also configured to reduce power consumption regarding the energy efficiency of 6G networks. Overall, the GFB-OFDM provides a versatile and efficient solution for the complex demands of 6G communication, particularly in supporting ultra-wideband THz communication, improving spectral efficiency, and offering adaptable configurations for diverse applications and services.

## 2. GFB-OFDM Transceiver

We first introduce the GFB-OFDM transmitter, and then the polyphase filter and GFB-OFDM receiver are illustrated, respectively. Finally, we provide the theoretical derivation.

### 2.1. Transmitter

**Figure 1:**
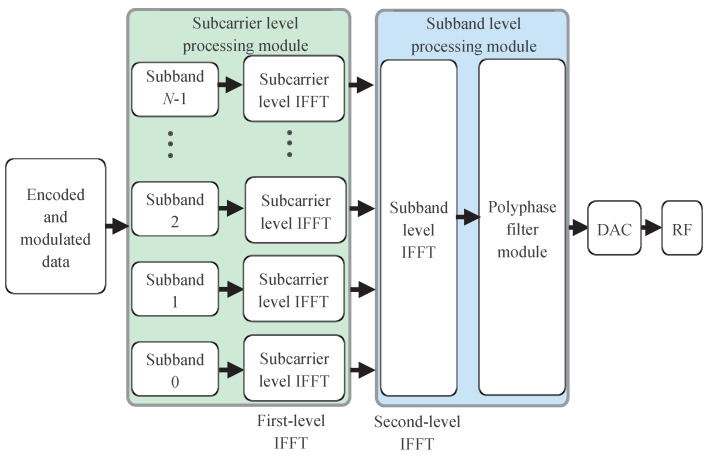
GFB-OFDM transmitter diagram. The encoded and modulated data are first divided into *N* subbands, with each subband undergoing a subcarrier-level IFFT. The resulting data is then processed through a subband-level IFFT along the subband dimension, followed by a polyphase filter. The output is transmitted after passing through the digital-to-analog converter and radio frequency module.

The GFB-OFDM transmitter is briefly exhibited in Figure 1. The modulated symbols are divided into *N* groups, and the symbols in each group are loaded onto its corresponding subband. Then, the first-level (or subcarrier-level) IFFT is performed over each individual subband, and the size of the IFFT is flexible, which aligns with the number of the loaded symbols in the specific subband. The output of each IFFT process is stacked, and the second-level (or subband-level) IFFT is performed over all the subband outputs; thus, the size of the second-level IFFT is the sum of the sizes of all subband IFFT. Then, the polyphase filter is adopted to window the baseband signal. Finally, the digital-to-analog converter (DAC) and the radio frequency (RF) is employed to transmit it out. In summary, the process of dividing modulated symbols into subbands and performing the first-level IFFT is referred to as the subcarrier-level processing module. The process of the second-level IFFT and the polyphase filter are referred to as the subband-level processing module.

By using the two levels of IFFT and the polyphase filters, the large size of the IFFT can be decomposed into two levels of IFFT of smaller sizes. This approach effectively addresses the challenges in the issues of the computational complexity and the hardware implementation.

In the following, we formulate the two-level IFFT and the polyphase filtering in the GFB-OFDM transmitter. There are *K* modulated symbols to be transmitted over the *K* subcarriers. They are divided into *N* subbands, and each subband has an identical bandwidth (each subband is able to alter its subcarrier spacing according to different services. For a brief illustration, we assume that each subband has identical subcarrier spacing.). In each subband, *M* symbols are mapped to the subcarrier, i.e., K=MN, which can be represented as X[l,k],l=0,1,2,⋯,M−1, k=0,1,2,⋯,N−1. Then, the output of the first-level IFFT x[m,k] of *N* subbands is calculated as

(1) $$x\left[m,k\right]=\frac{1}{M}\sum_{l=0}^{M-1}X\left[l,k\right]e^{j2\pi \frac{ml}{M}},\;m=0,1,2,\cdots ,M-1$$

where $j=\sqrt{-1}$. Let A be the inverse Fourier transform matrix; then, Equation (1) can be rewritten in the matrix form as

(2) $$x_{M\times N}=\frac{1}{M}A_{M\times M}X_{M\times N}.$$

Then, the cyclic prefix is added for each subband as

(3) $$x_{M\times N}\overset{\mathrm{Add}\;\mathrm{CP}}{\to }x_{(N_{\mathrm{cp}}+M)\times N}$$

where $N_{\mathrm{cp}}$ is the length of the cyclic prefix. Then, the second-level IFFT is performed row-wise over the matrix x. For each row, *N* samples are used as the input of the $N_{2}$ points double bandwidth oversampled IFFT, i.e., $N_{2}=2N$, and the input is given as

(4) $$Y_{\left(N_{\mathrm{cp}}+M\right)\times N_{2}}=\left[\begin{array}{c} 0_{\left(N_{\mathrm{cp}}+M\right)\times N_{s}},x_{(N_{\mathrm{cp}}+M)\times N},0_{\left(N_{\mathrm{cp}}+M\right)\times N_{s}} \end{array}\right]$$

where $N_{s}=\frac{N_{2}-N}{2}$ is the number of zero padding on one side. The operation is equivalent to oversampling in the time domain. After the $N_{\mathrm{cp}}+M$ second-level IFFT, the $N_{\mathrm{cp}}+M$ rows are obtained, and each row contains $N_{2}$ samples. Each row of a sample is a subsymbol data sequence, and the time-domain length *T* of a subsymbol data sequence is the reciprocal of the interval between the adjacent subband in the frequency domains, which is also the reciprocal of the width of the subband in the frequency domain. The second-level IFFT process can be written in matrix form as

(5) $$y_{\left(N_{\mathrm{cp}}+M\right)\times N_{2}}=\frac{1}{N_{2}}Y_{\left(N_{\mathrm{cp}}+M\right)\times N_{2}}B_{N_{2}\times N_{2}}$$

where B is the inverse Fourier transform matrix. The output of the second-level IFFT y is repeated *L* times column-wise, which is given as

(6) $$y_{\left(N_{\mathrm{cp}}+M\right)\times N_{2}}\overset{\mathrm{Repeat}\;L\;\mathrm{times}}{\to }y_{\left(N_{\mathrm{cp}}+M\right)\times LN_{2}}$$

Then, the output is filtered by the polyphase filter g, which is written as

(7) $$z_{\left(N_{\mathrm{cp}}+M\right)\times LN_{2}}=y_{\left(N_{\mathrm{cp}}+M\right)\times LN_{2}}⊙{\left[g,g,\cdots ,g\right]}_{\left(N_{\mathrm{cp}}+M\right)\times LN_{2}}$$

where “⊙” is the Hadamard product operation. The column vector g is the discrete form of the polyphase filtering function with the length of $LN_{2}$. Since each column of y is filtered by the same g, the filtering matrix is obtained by repeating g in $N_{\mathrm{cp}}+M$ times row-wise.

**Figure 2:**
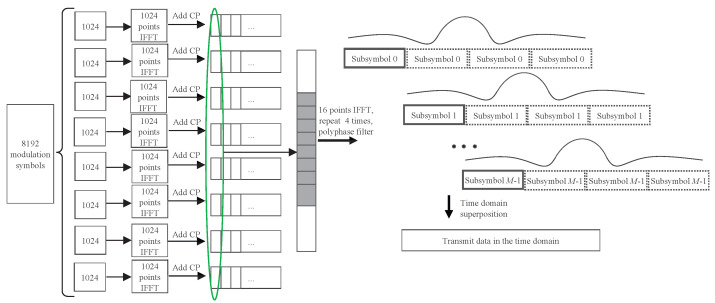
GFB-OFDM data-processing procedure with the number of modulated symbols K=8192, the number of subbands N=8 and the number of subcarriers in each subband M=1024.

Then, the $N_{\mathrm{cp}}+M$ subsymbol is summed over and the *m*th subsymbol is placed with the lag of the N(m−1) samples. The sum can be elaborated by Figure 2. Finally, the sum of the time-domain samples can be transmitted. Thus, the overall number of samples is $K+N(2L+N_{\mathrm{cp}}-1)$. In contrast, if the frequency-domain data symbols at the *K* subcarriers are transmitted in practice using the traditional CP-OFDM waveform, it generally requires at least $N_{\mathrm{ifft}}$-point IFFT, where $N_{\mathrm{ifft}}=2K$.

The aforementioned procedure demonstrates that GFB-OFDM can accomplish the IFFT of $N_{\mathrm{ifft}}$ points by utilizing *N* first-level IFFTs of *M* points and $N_{\mathrm{cp}}+M$ second-level IFFTs of $N_{2}$-points, thereby enabling the realization of a large-point IFFT through two small-point IFFTs. Moreover, the number of subbands and the number of subcarrier in each subband can be chosen according to the required traffic on demand.

### 2.2. Polyphase Filter

The purpose of the polyphase filter is to filter each subband in order to eliminate the mirror interference between the subbands. The parameters of the filtering function need to be selected carefully, considering its impact on the overall performance of GFB-OFDM [23,24]. In this paper, we adopt a dual root-raised-cosine (DRRC) filtering function, which employs the time-domain representation of the RRC function to window and truncate frequency-domain RRC signals. The procedure of generating this filter is as follows.

The time-domain form of the frequency-domain RRC function can be expressed as

(8) $$p\left(t\right)=\frac{\left(1-\alpha \right)T^{2}\sin c\left(\frac{t}{T}\left(1-\alpha \right)\right)+\frac{4}{\pi }\alpha T^{2}\cos\left(\frac{\pi t}{T}\left(1+\alpha \right)\right)}{T^{2}-16\alpha^{2}t^{2}}$$

where α is the roll-off factor of the RRC filter and *T* is the length of the subsymbol mentioned earlier, which means that the half-value bandwidth of this frequency domain RRC function is $\frac{1}{T}$, where $\frac{1}{T}$ represents the subband frequency domain bandwidth.

The time-domain RRC function is employed to truncate the temporal extent of the filter function. The time-domain RRC function is expressed as

(9) $$q\left(t\right)=\left\{\begin{array}{cc} 1, & 0\leq \left|t\right|<T_{b}\left(1-\beta \right)\\ \sqrt{\frac{1}{2}\left(1+\cos\left(\frac{\left|t\right|-T_{b}\left(1-\beta \right)}{2T_{b}\beta }\pi \right)\right)}, & T_{b}\left(1-\beta \right)\leq \left|t\right|<T_{b}\left(1+\beta \right)\\ 0, & \left|t\right|\geq T_{b}\left(1+\beta \right) \end{array}\right.$$

where β is the roll-off factor of the RRC function and $T_{b}$ is half of the time-domain bandwidth of the RRC function. Therefore, the length of this time-domain RRC function is $2T_{b}\left(1+\beta \right)$. Then, the time domain form g(t) of the DRRC filter is expressed as

(10) g(t)=q(t)p(t).

The time-domain length of the filtering function is equal to the length obtained by repeating each subsymbol in *L* times, i.e., $2T_{b}\left(1+\beta \right)=LT$. As *L* increases, which means the time-domain length of the filtering function becomes larger, the filtering function approaches an ideal frequency-domain RRC function. This enables an ideal elimination of interference between the subbands.

### 2.3. Receiver

**Figure 3:**
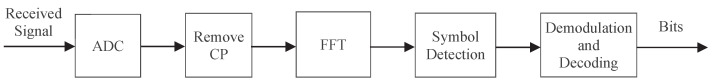
Processing at the receiver. The received time-domain signal is first converted into a digital signal via an ADC, followed by the removal of the cyclic prefix. After undergoing an FFT, the signal is converted to the frequency domain. In the frequency domain, it passes through the symbol detection module, as well as the demodulation and decoding module, resulting in the output information bits.

The detection of CP-OFDM can be directly used as the receiver of GFB-OFDM, as illustrated in Figure 3, which is validated by Theorem 1.

**Theorem 1.**  *For a signal of length NM, the following two operations result in exactly the same output. The first operation: perform a NM-point IFFT; the second operation: perform NM-point IFFTs, MN-point IFFTs and polynomial filtering.*

**Proof.**  Consider that X[l,k], consisting of NM information-bearing symbols, are transmitted on *M* subcarriers and *N* subbands. After performing first-level IFFT on *N* subbands, the data x[m,k] are obtained as

(11) $$x\left[m,k\right]=\frac{1}{M}\sum_{l=0}^{M-1}X\left[l,k\right]e^{j2\pi \frac{ml}{M}}$$

where $j=\sqrt{-1}$, m=0,1,2,⋯,M−1. l=0,1,2,⋯,M−1 and k=0,1,2,⋯,N−1 denotes the index of subcarriers and subbands, respectively. Perform *M* times of second-level IFFTs on *N* points, resulting in y[m,n], which can be expressed as

(12)y[m,n]=1N∑k=0N−1x[m,k]ej2πnkN(13)=1N1M∑k=0N−1∑l=0M−1X[l,k]ej2πmlMej2πnkN(14)=1NM∑k=0N−1∑l=0M−1X[l,k]ej2π(mlM+nkN).

For each subsymbol, repeat it *L* times to yield $y[m,n_{\mathrm{ifft}}]$, where $LN=N_{\mathrm{ifft}}$ and $n_{\mathrm{ifft}}=0,1,2,\cdots ,N_{\mathrm{ifft}}-1$.

(15) $$y\left[m,n_{\mathrm{ifft}}\right]=\frac{1}{\sqrt{N_{\mathrm{ifft}}}}\sum_{k=0}^{N-1}\sum_{l=0}^{M-1}X\left[l,k\right]e^{j2\pi (\frac{ml}{M}+\frac{n_{\mathrm{ifft}}k}{N})}.$$

Here, the analysis should be conducted from a frequency domain perspective. Therefore, an FFT is performed on each symbol to obtain the frequency domain information $Y[m,n_{\mathrm{ifft}}]$, which is expressed as

(16)Y[m,nifft]=1Nifft∑ni=0Nifft−1y[m,ni]e−j2πninifftNifft(17)=1Nifft1Nifft∑ni=0Nifft−1∑k=0N−1∑l=0M−1X[l,k]ej2π(mlM+nikN)e−j2πninifftNifft(18)=1Nifft∑k=0N−1∑l=0M−1X[l,k]ej2πmlM∑ni=0Nifft−1ej2π(nikN−ninifftNifft)(19)=1Nifft∑k=0N−1∑l=0M−1X[l,k]ej2πmlM∑ni=0Nifft−1ej2πnikM−nifftNifft(20)=∑k=0N−1∑l=0M−1X[l,k]ej2πmlMδ[(kM−nifft)Nifft].

where “${\left[\;\cdot \;\right]}_{N_{\mathrm{ifft}}}$” denotes the modulo $N_{\mathrm{ifft}}$ operation and δ[·] denotes the Dirac delta function. We can the obtain

(21) $$Y\left[m,n_{\mathrm{ifft}}\right]=\left\{\begin{array}{cc} \sum_{l=0}^{M-1}X\left[l,\frac{n_{\mathrm{ifft}}}{M}\right]e^{j2\pi \frac{ml}{M}}, & n_{\mathrm{ifft}}=kM\\ 0, & n_{\mathrm{ifft}}\neq kM. \end{array}\right.$$

Then, Y[m,p] is convolved with the rectangular filter FIL[p]. At the mean time, the temporal misalignment process is transformed into a phase rotation in the frequency domain. Then, we can obtain

(22)W[m,b]=Y[m,p]⊗FIL[p]e−j2πmNNifftb(23)=∑p=0Nifft−1Y[m,p]|FIL[b−p]|e−j2πmNNifftb(24)=∑p=0p=kMNifft−1∑l=0M−1X[l,pM]ej2πmlM|FIL[b−p]|e−j2πmNNifftb(25)=∑p=0p=kMNifft−1∑l=0M−1X[l,pM]|FIL[b−p]|ej2πml−bM

where $b=0,1,2,\cdots ,N_{\mathrm{ifft}}-1$ and $p=0,1,2,\cdots ,N_{\mathrm{ifft}}-1$. “⊗” and “|·|” denotes the circular convolution symbol and the module value, respectively. FIL[p]=0 when p≥M, and $\mathrm{FIL}\left[p\right]=\frac{1}{M}$ when 0≤p≤M−1. Sum the frequency domain data of different subsymbols to obtain

(26)Z[b]=∑m=0M−1W[m,b](27)=∑p=0p=kMNifft−1∑l=0M−1X[l,pM]|FIL[b−p]|∑m=0M−1ej2πml−bM(28)=M∑p=0p=kMNifft−1∑l=0M−1X[l,pM]|FIL[b−p]|δ[(l−b)M](29)=M∑p=0p=kMNifft−1X[(b)M,pM]|FIL[b−p]|(30)=M∑k=0N−1X[(b)M,k]|FIL[b−kM]|(31)=X[(b)M,⌊bM⌋]

where ⌊·⌋ denotes the rounding down operation. At this time, it means that the modulation symbols are completely recovered from the data Z[b]. □

The receiver first removes the cyclic prefix of each received OFDM symbol, followed by the FFT to recover the modulated symbols. Subsequently, the symbol detection, the demodulation, and the decoding are performed to ultimately retrieve the information elements. Note that the number of repetition *L* affects the inference between the subsymbols and the one between the subbands. That is, as *L* increases, the interference between the subsymbols becomes larger, the interference between the subbands becomes smaller, and the computational complexity of Equation (7) becomes higher. To reduce the complexity and the overhead of the cyclic prefix, a small *L* is preferred; however, it may introduce the interference between the subcarriers. The simulation results in Section 3 show that the interference between the subbands is tolerable when L≥4, and the interference between the subsymbols can be ignored. In this case, the receiver can receive in an almost lossless manner.

### 2.4. Receiver Analysis and Its Extension

**Figure 4:**
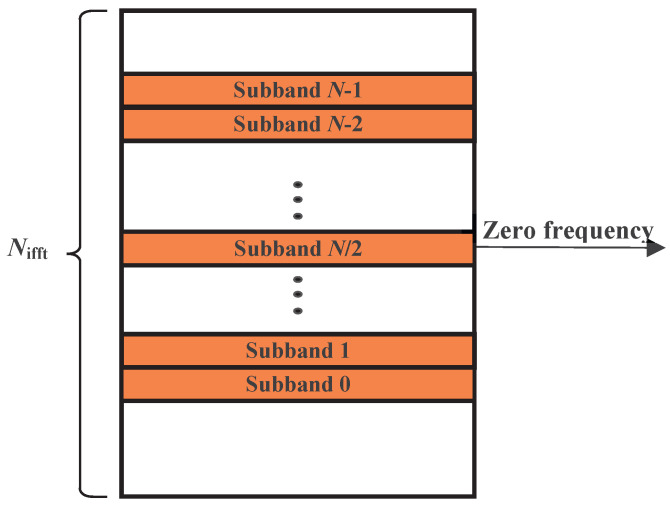
The locations of the *N* subbands in the frequency domain.

In this section, we discuss a special case of Theorem 1 where oversampling and different filter lengths are considered. Similar to previous discussions, the total number of IFFT points is $N_{\mathrm{ifft}}$ and the data bandwidth is divided into *N* subbands as shown in Figure 4, each with *M* subcarriers. $N_{2}$ represents the second-level IFFT point number, where $N_{\mathrm{ifft}}$, *M*, and $N_{2}$ are all positive integer power of two. The length of the filter is $LN_{2}$.

Firstly, let us analyze the case where $LN_{2}=N_{\mathrm{ifft}}$ for filter length selection. Due to the characteristic of orthogonal frequency domain subcarriers, we only need to track the process experienced by data on one subcarrier in the GFB-OFDM waveform at its transmission end in order to easily extend it to the transmission processes of all subcarriers. Assuming that one data *d* is transmitted on subcarrier *P* in subband *Q*, then we can analyze the transmission data processing of GFB-OFDM according to the following steps:

(1) The data $X_{M\times N}$ across *N* subbands and *M* subcarriers can be expressed as
  (32) $$X\left[m,n\right]=\left\{\begin{array}{cc} d, & m=P\;\mathrm{and}\;n=Q\\ 0, & m\neq P\;\mathrm{or}\;n\neq Q. \end{array}\right.$$
(2) After performing first-level IFFT on *N* subbands, the data $x_{M\times N}$ is obtained as
  (33) $$x\left[m,n\right]=\mathrm{IFFT}\left(X\left[m,n\right]\right)=\left\{\begin{array}{cc} \frac{d}{\sqrt{M}}e^{j2\pi \frac{Pm}{M}}, & n=Q\\ 0, & n\neq Q. \end{array}\right.$$
(3) Perform *M* times of second-level IFFTs on $N_{2}$ points, resulting in $y_{M\times N_{2}}$. The position of the subbands determines the mapping location for the data in the second-level IFFT, where $n_{2}=0,1,2,\cdots ,N_{2}-1$. Then, $y_{M\times N_{2}}$ can be expressed as
  (34) $$\begin{array}{cc} y[m,n_{2}]\; & =\frac{d}{\sqrt{N_{\mathrm{ifft}}}}e^{j2\pi (\frac{Pm}{M}+n_{2}\frac{N_{s}+Q}{N_{2}})}. \end{array}$$
(4) For each subsymbol, repeat it *L* times to yield $y_{M\times LN_{2}}$. According to this assumption, $LN_{2}=N_{\mathrm{ifft}}$ can be written as $y_{M\times N_{\mathrm{ifft}}}$, where $n_{\mathrm{ifft}}=0,1,2,\cdots ,N_{\mathrm{ifft}}-1$.
  (35) $$y\left[m,n_{\mathrm{ifft}}\right]=\frac{d}{\sqrt{N_{\mathrm{ifft}}}}e^{j2\pi (\frac{Pm}{M}+n_{\mathrm{ifft}}\frac{N_{s}+Q}{N_{2}})}.$$
(5) Here, the analysis should be conducted from a frequency domain perspective because it is believed that the frequency domain information after undergoing second-level IFFT should still contain the information from the original subcarriers. Therefore, an FFT is performed on each symbol to obtain the frequency domain information $Y_{M\times N_{\mathrm{ifft}}}$, which is expressed as
  (36)Y[m,nifft]=FFT(yM×Nifft)(37)=dNifft∑ni=0Nifft−1ej2π(PmM+niNs+QN2)1Niffte−j2πninifftNifft(38)=dNifft∑ni=0Nifft−1ej2πPmMej2πni((Ns+Q)MN2M−nifftNifft).
  We can obtain
  (39) $$Y\left[m,n_{\mathrm{ifft}}\right]=\left\{\begin{array}{cc} de^{j2\pi \frac{Pm}{M}}, & n_{\mathrm{ifft}}=\left(N_{s}+Q\right)M\\ 0, & n_{\mathrm{ifft}}\neq \left(N_{s}+Q\right)M. \end{array}\right.$$
(6) $Y_{M\times N_{\mathrm{ifft}}}$ is convolved with the rectangular filter FIL. At the mean time, the temporal misalignment process is transformed into a phase rotation in the frequency domain. Then, we can obtain
  (40)W[m,b]=Y[m,(Ns+Q)M]⊗FILe−j2πmN2Nifftb(41)=d|FIL[b]|ej2πPmMe−j2πmN2Nifftb(42)=d|FIL[b]|ej2πmP−bM
  where $b=0,1,2,\cdots ,N_{\mathrm{ifft}}-1$ represents subcarriers in the frequency domain, “⊗” is the circular convolution symbol, and “|·|” denotes the module value.
(7) Sum the frequency domain data of different subsymbols to obtain $Z_{N_{\mathrm{ifft}\times 1}}$
  (43)Z[b]=∑m=0M−1W[m,b](44)=d|FIL[b]|∑m=0M−1ej2πmP−bM.

Since the purpose of polyphase filtering is to filter data of each subband separately, it can be understood that the frequency domain carrier positions corresponding to the passband bandwidth of the filter are $b=\left[\left(N_{s}+Q\right)M-\frac{M}{2},\;\left(N_{s}+Q\right)M+\frac{M}{2}-1\right]$. It should be noted that the positions of the filters differ for different subbands. Additionally, because we have normalized the peak value of our polyphase filtering filters in the time domain, and because their frequency domain bandwidth corresponds to the subband width, we can obtain the frequency domain coefficients of these filters.

(45) $$\left|\mathrm{FIL}\left[b\right]\right|=\frac{1}{M}.$$

According to (40), it can be similarly derived that, when $b=\left(N_{s}+Q\right)M-\frac{M}{2}+P$, we have

(46) $$Z\left[\left(N_{s}+Q\right)M-\frac{M}{2}+P\right]=d.$$

At this time, it means that the data on this subcarrier are represented as *d*, while the data on other subcarriers are zero. The time-domain information corresponding to $Z_{N_{\mathrm{ifft}}\times 1}$, denoted as $z_{N_{\mathrm{ifft}}\times 1}$, represents the time-domain data after performing IFFT. At the receiver, CP-OFDM can be used to receive data in the frequency domain, and the data on each subcarrier satisfies orthogonality. This is consistent with the conclusion of Theorem 1.

More generally, when the length of the filter is $L(1\leq L\leq \frac{N_{\mathrm{ifft}}}{N_{2}})$ and it is a DRRC filter with double roots, (35) becomes

(47) $$y\left[m,n_{\mathrm{ifft}}\right]=\left\{\begin{array}{cc} \frac{d}{\sqrt{N_{\mathrm{ifft}}}}e^{j2\pi (\frac{Pm}{M}+n_{\mathrm{ifft}}\frac{N_{s}+Q}{N_{2}})}, & n_{\mathrm{ifft}}<LN_{2}\\ 0, & n_{\mathrm{ifft}}\geq LN_{2}. \end{array}\right.$$

Assuming the filter coefficients of $LN_{2}$ points, the total time-domain filter coefficients for one OFDM symbol length are given by $\mathrm{fil}=\left[\mathrm{fil}0,\mathrm{zeros}(N_{\mathrm{ifft}}-LN_{2},1)\right]$. Then, the point-wise multiplication result of the symbol *m* is subjected to FFT and symbol shifting and we can obtain

(48)W[m,b]=FFT(ym,nifft×fil(nifft))e−j2πmN2Nifftb(49)=dej2π(P−b)mMFFT(ej2πMnifftNs+QMN2×fil(nifft))(50)=dej2π(P−b)mMFIL(b−M(Ns+Q)Nifft)(51)=dej2π(P−b)mM|FIL(b−M(Ns+Q)Nifft)|

It should be noted that the peak value of the filter in the time domain occurs at t=0 and that its frequency-domain coefficients are real numbers. The data of different subsymbols are then superimposed in the frequency domain.

(52)Z[b]=∑m=0M−1W[m,b](53)=d|FIL(b−M(Ns+Q)Nifft)|∑m=0M−1ej2πmP−bM.

It is easy to determine that the passband of the filter is $b=[\left(N_{s}+Q\right)M-\frac{M}{2},\left(N_{s}+Q\right)M+\frac{M}{2}-1]$, so Z[b] only has a value when $b=\left(N_{s}+Q\right)M-\frac{M}{2}+P$, which is

(54) $$Z\left[\left(N_{s}+Q\right)M-\frac{M}{2}+P\right]=d\left|\mathrm{FIL}\left({\left[P-\frac{M}{2}\right]}_{N_{\mathrm{ifft}}}\right)\right|.$$

It effectively filters the data on the original subcarrier while setting the information on other subcarriers to zero, achieving the effect of a two-level IFFT conversion with a first-level IFFT and filtering. Each subband is filtered individually, and then all different subbands are superimposed in the time domain.

### 2.5. Advantages

GFB-OFDM utilizes the two-level IFFTs to decompose the original large-size IFFT into two small-size IFFTs, enabling the operations of large-size IFFT dedicated to the ultra-wide bandwidth scenarios. Furthermore, the GFB-OFDM waveform possesses the following advantages:

(1) By alternating the number of subbands while fixing the subband bandwidth, it is possible to achieve data services in different channel bandwidths. Since the filtering is performed on each subbband, a single filter is sufficient. In contrast, each service with a different bandwidth required a different filter in CP-OFDM. This facilitates the flexible support for various channel bandwidth requirements in certain hardware scenarios.
(2) The polyphase filtering in GFB-OFDM efficiently employs an individual filter for each subband, thus mitigating the potential occurrence of the out-of-band leakage of the subbands. Therefore, it can accommodate various scenarios where different subcarrier spacings are used for different subbands, and these diverse subcarrier spacings can be uniformly processed using GFB-OFDM waveform processing.
(3) Different types of data can be assigned to different subbands within a single GFB-OFDM symbol, and they can be modulated either in a multicarrier manner or a single-carrier manner. The submodule for per subband processing in GFB-OFDM enables a simultaneous processing of different types of data-bearing subbands, achieving an integration of single-carrier processing and the multi-carrier processing.
(4) The receiver of the 4G/5G OFDM is the legacy of the proposed GFB-OFDM waveform without introducing any additional complexity.

## 3. Simulation Results

In this section, the performance evaluation and the analysis of the GFB-OFDM and the CP-OFDM of 5G NR are provided. In Table 1, it shows the simulation parameter setup. It is assumed that the CP-OFDM waveform can support an IFFT of 16,384-point (double bandwidth oversampled IFFT).

### 3.1. Out-Of-Band Leakage

**Figure 5:**
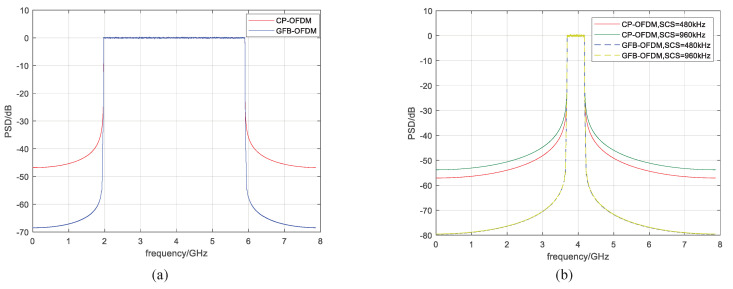
The out-of-band leakage of CP-OFDM and GFB-OFDM is evaluated. Specifically, the number of the subbands are 8 and 1 in the corresponding (**a**,**b**). The results demonstrate that the out-of-band leakage of GFB-OFDM is lower than the OFDM because of the polyphase filter operation.

The power spectral density (PSD) of CP-OFDM and GFB-OFDM are shown in Figure 5, with eight subbands and one subband chosen, respectively, as in Table 1. Due to the implementation of the polyphase filtering, the GFB-OFDM exhibits lower out-of-band leakage compared to CP-OFDM, as evident from the results in Figure 5a. This reduction in the out-of-band leakage allows for a decrease in the guard bandwidth and an improvement in the spectral efficiency. In Figure 5b, the out-of-band leakage of one subband is depicted, which reflects the inter-subband interference.

It can be observed that the out-of-band leakage of one subband is significantly higher than that of GFB-OFDM. Specifically, the subband with a 960 kHz subcarrier spacing demonstrates heavier out-of-band leakage than that with a 480 kHz subcarrier spacing. Conversely, in GFB-OFDM, the out-of-band leakage across different subcarrier spacings remains consistently low.

### 3.2. BLER in Same Subcarrier Spacing

**Figure 6:**
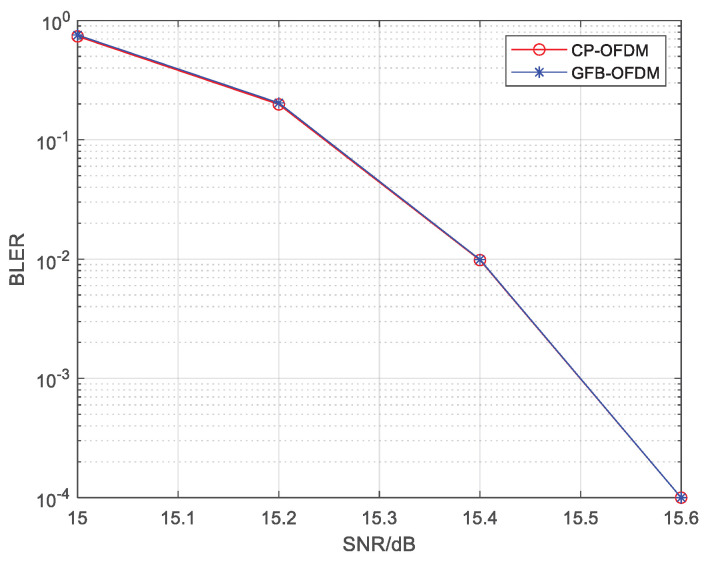
The The BLER of CP-OFDM and GFB-OFDM is evaluated for the same subcarrier spacing scenario. The rate-$\frac{3}{4}$ LDPC channel coding, the 64QAM modulation and the AWGN channel are employed. The result demonstrates that GFB-OFDM maintains a nearly identical BLER performance compared to CP-OFDM.

Let all subcarriers in the channel bandwidth have an identical subcarrier spacing of 480 kHz. In order to reduce the interference between subbands in GFB-OFDM, the first-level IFFT is oversampled with the double bandwidth, i.e., a 2048-point IFFT. Traditional CP-OFDM is adopted. Figure 6 presents the BLER of CP-OFDM and GFB-OFDM, showing that the BLER performance of the two waveforms are almost the same. This result confirms that the inter-subband interference can be ignored and that there is no degradation in the BLER of GFB-OFDM when L≥4.

### 3.3. BLER in Different Subcarrier Spacing

**Figure 7:**
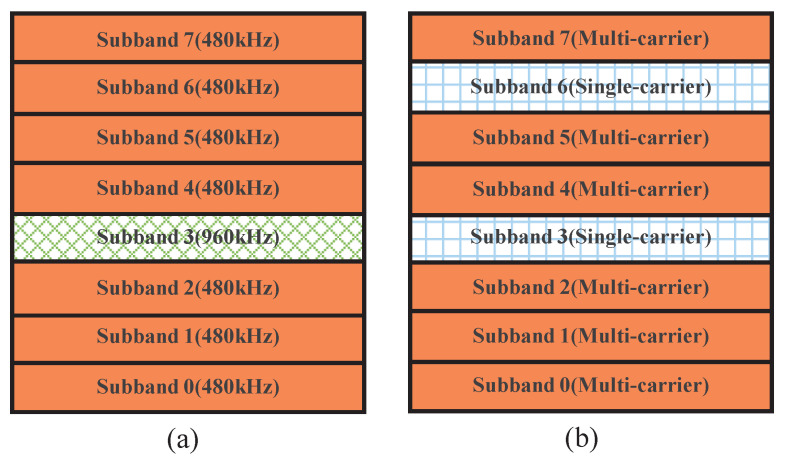
The locations of the eight subbands in the frequency domain under two cases. (**a**) represents the mixture scenarios of different subcarrier spacings, which were 480 kHz and 960 kHz, respectively. (**b**) represents single-carrier and multi-carrier mixture scenarios.

**Figure 8:**
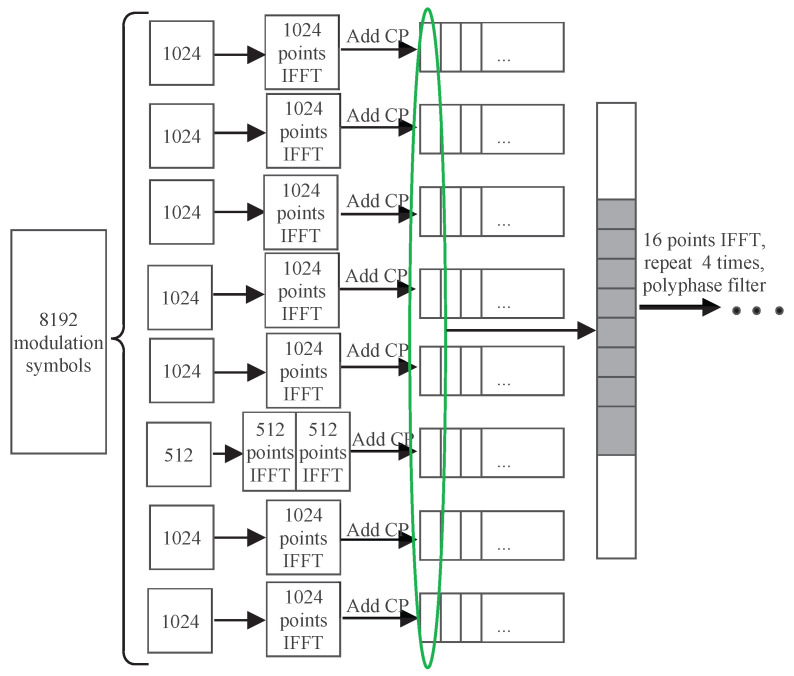
Subband data-processing procedure for different subcarrier spacing with the number of modulated symbols K=8192, the number of subbands N=8, and the number of subcarriers in each subband M=1024 or M=512.

When different subcarrier spacings exist within the channel bandwidth, a coexistence case of the subbands with different subcarrier spacings is shown in Figure 7a, where Subband 3 has a subcarrier spacing of 960 kHz while the rest have a spacing of 480 kHz. The traditional CP-OFDM cannot process the data from different subbands together during the IFFT. However, the proposed GFB-OFDM can handle all subbands together, demonstrating flexibility for various requirements. The specific procedure is illustrated in Figure 8. Specifically, after performing the first-level IFFT over the adjacent symbols of Subband 3 separately and then adding CP, they are concatenated and then serve as the input of the second-level IFFT along with the other subbands. In this simulation, the CP-OFDM waveform is obtained by the oversampled IFFT operations on the data with a subcarrier spacing of 480 kHz and 960 kHz, respectively, using 16,384 points. Subsequently, the two sets of the time-domain data are combined into one set.

**Figure 9:**
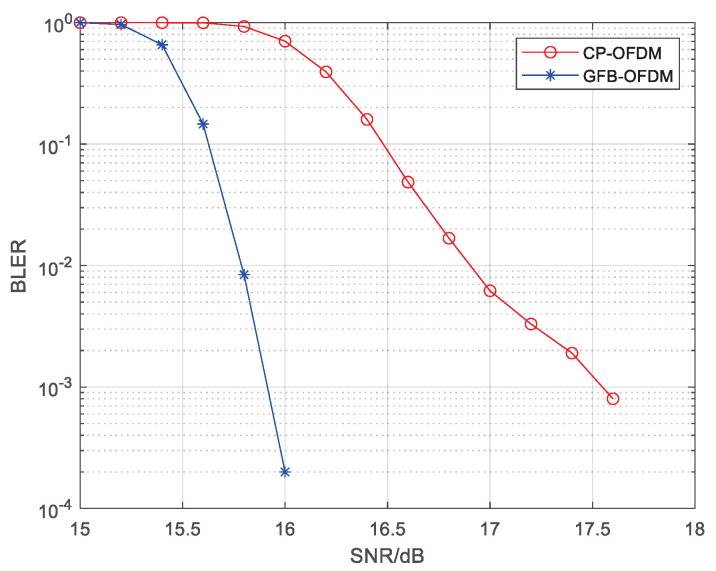
The BLER of CP-OFDM and GFB-OFDM is evaluated in different subcarrier spacing scenarios. The rate-$\frac{3}{4}$ LDPC channel coding, the 64 QAM modulation, and the AWGN channel are employed. Specifically, subbands with subcarrier spacings of 480 kHz and 960 kHz coexist. The result demonstrates that the BLER performance of GFB-OFDM is significantly superior to that of CP-OFDM.

Figure 9 presents a comparison of the BLER performance in demodulating data transmitted on subband 3. Due to the interference from the other subbands with different subcarrier spacings, the BLER performance of subband 3 degrades. It can be also observed that GFB-OFDM outperforms CP-OFDM since GFB-OFDM employs the polyphase filtering, resulting in low out-of-band leakage for each subband and thus smaller inter-subband interference compared to the CP-OFDM. Therefore, GFB-OFDM outperforms CP-OFDM in BLER in the different subcarrier spacing scenario, which facilitates a dynamic and flexible adjustment scheme for the subcarrier spacing between the subbands in regard to 6G application.

### 3.4. BLER in Mixture Scenarios of Single-Carrier and Multi-Carrier

**Figure 10:**
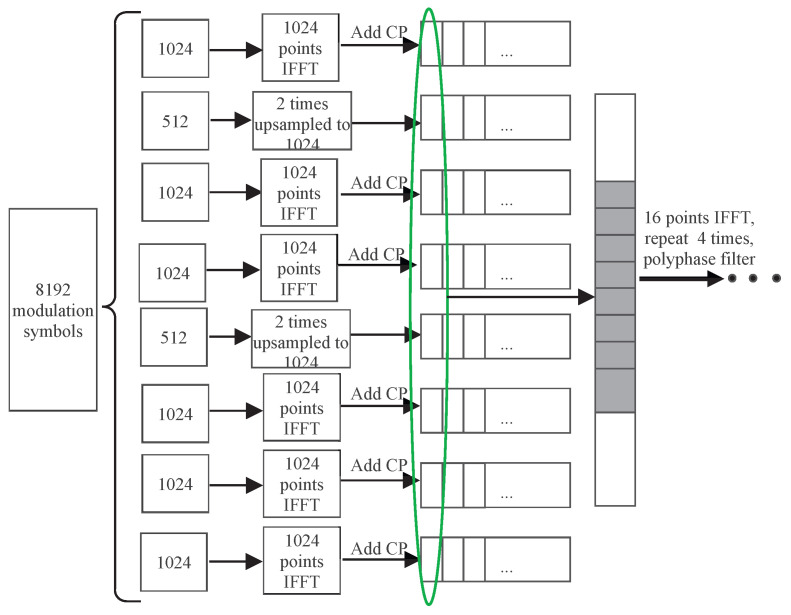
Subband data-processing procedure for a single-carrier and multi-carrier mixture scenario with the number of modulated symbols K=8192, the number of subbands N=8 and the number of subcarriers in each subband M=1024 or M=512.

In the mixture scenario of the single-carrier and the multi-carrier modulation, as in Figure 7b, subband 3 and subband 6 are the single-carrier subbands; the remaining subbands are multi-carrier subbands with subcarrier spacings of 480 kHz which employ an oversampling IFFT operation with 16,384 points. CP-OFDM requires alternative processing for the single-carrier and the multi-carrier subbands, followed by time-domain superposition, with each single-carrier subband being sent individually. In contrast, the proposed GFB-OFDM scheme allows any subband to be used as either a single-carrier or a multi-carrier subband without increasing any complexity, enabling all single-carrier and multi-carrier subbands to be processed simultaneously. The specific process is illustrated in Figure 10.

**Figure 11:**
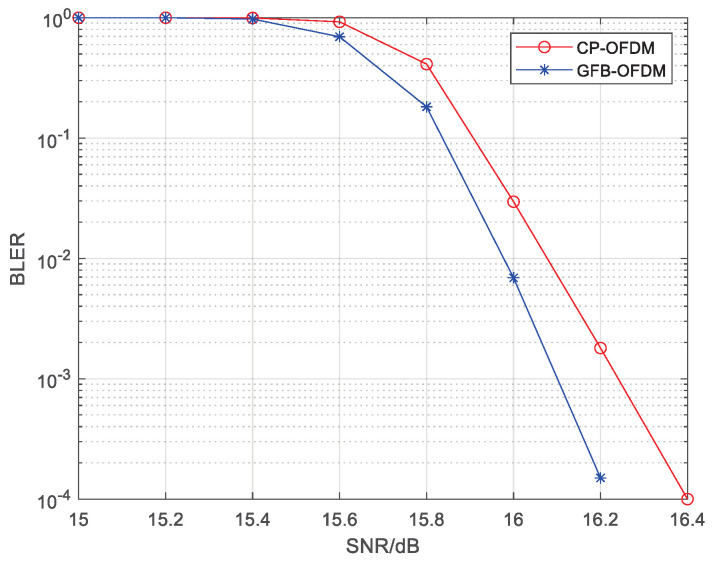
The BLER of CP-OFDM and GFB-OFDM is evaluated in single-carrier and multi-carrier mixture scenarios. The rate-$\frac{3}{4}$ LDPC channel coding, the 64 QAM modulation, and the AWGN channel are employed. The result demonstrates that the BLER performance of GFB-OFDM is superior to that of CP-OFDM.

Figure 11 presents a comparison of the BLER performance for demodulating the data transmitted on subband 3 at the receiver. Due to interference from other subbands, subband 3 experiences some degradation in terms of BLER performance. From the comparison results shown in the figure, it can be observed that GFB-OFDM outperforms CP-OFDM to some extent. This is mainly because GFB-OFDM has a polyphase filter effect at the transmitter, resulting in low out-of-band leakage for each subband and thus smaller inter-subband interference compared to CP-OFDM, leading to a better BLER performance. Therefore, in scenarios involving a mixture of single-carrier and multi-carrier transmissions, GFB-OFDM exhibits slightly better performance than CP-OFDM while maintaining lower processing complexity and greater flexibility.

### 3.5. Complexity Analysis

**Figure 12:**
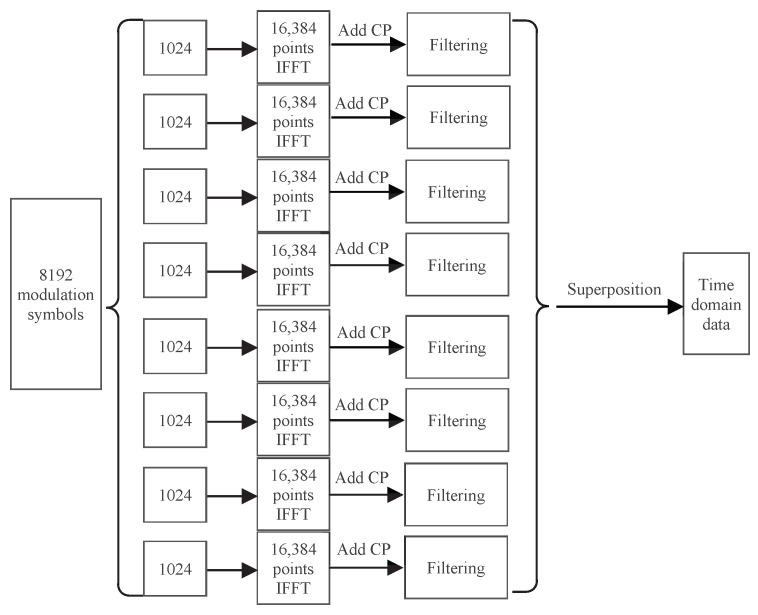
CP-OFDM data processing with filtering. The number of modulated symbols, subbands, and subcarriers are set as K=8192, N=8 and M=1024.

In general, the complexity of a multiplier is significantly higher than that of an adder. Hence, we focus on the comparison of the multiplication between CP-OFDM and GFB-OFDM. In addition, considering the flexibility in the subcarrier spacing adjustment, the CP-OFDM necessitates the inclusion of the filters for each subband. These filters are positioned differently to correspond to the frequency range of each subband respectively. Note that, since all subbands are the multi-carrier subbands, it illustrates the transmission process of CP-OFDM in Figure 12. Both GFB-OFDM and CP-OFDM require adding CP with a similar complexity. Thus, we can ignore the presence of CP during the comparison.

First, we analyze the number of multiplications involved in the IFFT and the filtering of GFB-OFDM. The GFB-OFDM employs the two-level IFFTs, where it can be deduced from the processing procedure that the first-level IFFT is of M points. Hence, the overall number of multiplications in *N* subbands for first-level IFFT processing is $\frac{NM}{2}\log M$ [25]. The number of multiplications in *M* second-level IFFT points is $\frac{N_{\mathrm{ifft}}}{M}$, so the total number of multiplications in *M* second-level IFFT is $\frac{N_{\mathrm{ifft}}}{2}\log\frac{N_{\mathrm{ifft}}}{M}$. In addition, the number of multiplications in the polyphase filtering process of GFB-OFDM can be determined using the following calculation. Considering that a second-level IFFT has $\frac{N_{\mathrm{ifft}}}{M}$ points, the length of time-domain data after IFFT is $\frac{N_{\mathrm{ifft}}}{M}$. Therefore, by repeating these data *L* times and performing point multiplication and windowing operations, the total number of multiplications becomes $\frac{LN_{\mathrm{ifft}}}{M}$. Consequently, for one OFDM symbol, the overall number of multiplications required for point multiplication and windowing operations is $\frac{M\times LN_{\mathrm{ifft}}}{M}=LN_{\mathrm{ifft}}$.

Hence, the total number of multiplications involved in IFFT and filtering processing for GFB-OFDM is as follows:

(55) $$O\left(\mathrm{GFB}-\mathrm{OFDM}\right)=\frac{NM}{2}\log M+\frac{N_{\mathrm{ifft}}}{2}\log\frac{N_{\mathrm{ifft}}}{M}+LN_{\mathrm{ifft}}.$$

We then analyze the number of multiplications involved in the IFFT and filtering process of CP-OFDM. The number of multiplications in the IFFT processing of CP-OFDM is $\frac{N_{\mathrm{ifft}}N}{2}\log N_{\mathrm{ifft}}$, which is determined based on $N_{\mathrm{ifft}}$-point IFFT with *N* subbands.

The calculation process for the number of multiplications in the time-domain convolution filtering of CP-OFDM is as follows. The number of time-domain data points for one OFDM symbol is $N_{\mathrm{ifft}}$, and the number of points included in the time-domain length of the filter function is $\frac{LN_{\mathrm{ifft}}}{M}$. Therefore, the total number of multiplications for *N* subbands processed by time-domain convolution filtering is $\frac{NLN_{\mathrm{ifft}}^{2}}{M}$. Hence, the total number of multiplications in the IFFT and filtering processing for CP-OFDM is

(56) $$O\left(\mathrm{OFDM}\right)=\frac{N_{\mathrm{ifft}}N}{2}\log N_{\mathrm{ifft}}+\frac{NLN_{\mathrm{ifft}}^{2}}{M}.$$

**Figure 13:**
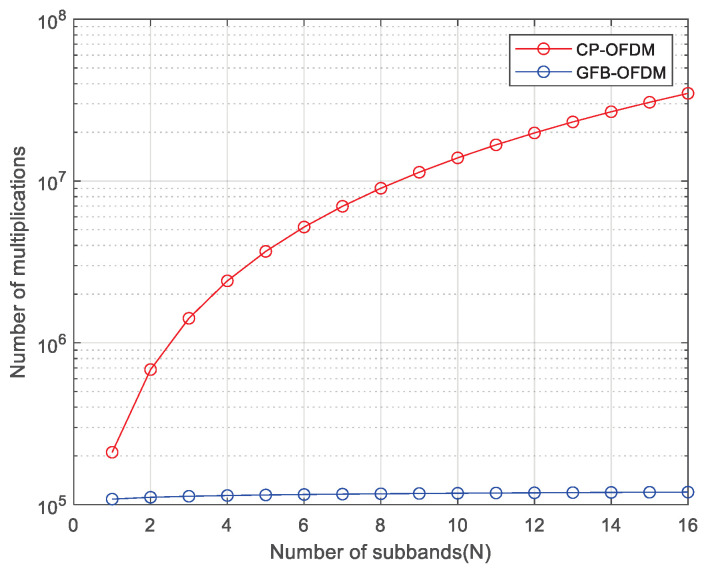
The complexity of CP-OFDM and GFB-OFDM is evaluated under a varying number of subbands. Under different numbers of subbands, the number of multiplications required by GFB-OFDM is less than that of CP-OFDM. In addition, as the number of subbands increases, the number of multiplications required by CP-OFDM increases rapidly. In contrast, the number of multiplications required by GFB-OFDM increases slowly.

The calculation process above ignores cyclic prefix because its length has a minimal impact on the overall complexity. It is easy to observe that (36) is much smaller than (37). Figure 13 illustrates the relationship between multiplication complexity and the number of subbands for two waveforms, assuming $N_{\mathrm{ifft}}=16,384$, L=4, the total number of subcarriers is 8192, and that it is divided into *N* subbands. It can be seen that GFB-OFDM has lower complexity compared to CP-OFDM and that the difference becomes more pronounced as the number of subbands N increases. For example, using the calculations in Section 3.2 as an example, the number of multiplications for transmitting one OFDM symbol in CP-OFDM and GFB-OFDM are approximately $9.0\times 10^{6}$ and $1.1\times 10^{5}$, respectively.

As previously mentioned, GFB-OFDM exhibits lower complexity compared to CP-OFDM due to the following reasons:

(1) In CP-OFDM, subbands with different subcarrier spacings require independent processing and are then combined in the time domain using IFFT, leading to increased computational complexity. As the number of subbands requiring separate processing grows, the overall complexity rises substantially.
(2) Conversely, GFB-OFDM enables uniform processing of subbands with different subcarrier spacings, providing greater flexibility in their division. Modifying the size or increasing the number of subband divisions does not augment processing complexity while maintaining a constant bandwidth.

## 4. Conclusions

In this paper, we proposed the new waveform GFB-OFDM, which could be a candidate for 6G communication. It utilizes two levels of IFFTs, both in small sizes, and adopts the polyphase filtering to attain the same effect of the corresponding IFFT in the large size, which supports wideband communication. By adjusting the number of subbands for a fixed subband bandwidth, it enables data transmission in different channel bandwidths and thus facilitates a flexible support for various service requirements regarding hardware constraints. It can further adapt to the scenarios with various subcarrier spacings for each subband and accommodate both the single-carrier modulation and the multi-carrier modulation without increasing any computational complexity. We then provided the analysis and the simulation, and they verify that GFB-OFDM demonstrates the aforementioned advantages in the THz high-bandwidth communication and in the varying subcarrier spacing requirements. In future 6G communications that cover numerous complex application scenarios, different performance indicators could vary according to the specific applications. The proposed GFB-OFDM is capable of supporting the characteristic requirements of ultra-wide bandwidth, ultra-high speed, different data services, different subcarrier spacings, and different channel bandwidths in various scenarios and demands.

## Figures and Tables

**Table 1:** Simulation Parameters.

Parameters	CP-OFDM	GFB-OFDM
Total number of subcarriers	8192	8192
Number of subbands	8	8
Number of subcarriers in each subband	1024	1024
Size of total IFFT	16,384	16,384
Size of first-level IFFT	/	2048
Size of second-level IFFT	/	16
Subcarrier spacing	480 kHz, 960 kHz	480 kHz, 960 kHz
Filter bandwidth	/	1 times the subband width
The length of the time domain filter	/	4 times the subsymbol length
Roll down factor α of frequency domain DRRC filter	/	0.15
Roll down factor β of the DRRC window filter	/	0.5
MCS	3/4 LDPC, 64 QAM	3/4 LDPC, 64 QAM
Length of CP	1/16 of the symbol length	1/16 of the symbol length
Channel	AWGN	AWGN

## Data Availability

Data are contained within the article.
